# The Case of Mr. Millar

**Published:** 1857-01-01

**Authors:** 


### THE CASE OF MR. MILLAR.

(*From the "Bucks Advertiser and Aylesbury News" of Oct.* 18, 1856.)

It is little use to declaim against absolute authority unless we can also show that it has been misused. The pomp and show of authority may be very agreeable, but the actual work of administering the affairs of a public institution—such, for example, as the County Lunatic Asylum—is by no means to be coveted, and any one who will undertake the work and *do it well,* deserves the public gratitude. Instead of discussing, on abstract grounds, whether the County Magistrates are the most proper persons to be entrusted with the management of the county funds, and (what is of much more consequence) the administration of justice, we shall prefer to notice, from time to time, the way in which these duties are actually discharged. If the system is found to answer tolerably well, he would be a bold, rather than a wise man, who would seek to uproot it. If, on the contrary, the management of a great portion of the public business turns out to be as bad as it well can be, then discretion would seem to point in the direction of change. For the present, our business is with facts.

The Lunatic Asylum at Stone is under the control of a committee of twelve magistrates, elected by the Court of Quarter Sessions. The medical superintendence, since the commencement, has been entrusted to Mr. John Millar. Of this gentleman, personally, we know next to nothing, and, of course, have no special interest in his affairs, nor can we decide, of our own knowledge, whether he is a fit person for such an office or not. All we know about him is derived from the reports which have from time to time been made public by the Committee, and certain other official documents made public in a letter from Mr. Millar, to which we recently invited attention. It is clearly on record, under the signature of most of the gentlemen who now stand forward as Mr. Millar's accusers, that up to a very recent period, Mr. Millar's conduct in the discharge of his duties has been not only unexceptionable, but such as to entitle him to the warmest thanks of the Committee. If there has been a single dissentient voice, it has never been publicly raised. Of course it is abstractedly possible that, in spite of all this, Mr. Millar may be the most unfit man in the world for his post. We confess we do not think much the better of him for having enjoyed the favour of such worthies as these Visiting Justices appear by their subsequent conduct to be. But whether he deserved his high character or not, he was entitled to the benefit of it, at least to this extent—that if any cause of complaint arose, a fair latitude of defence should be allowed him, and the best construction put upon any doubtful matters. Instead of this, it appears that Mr. Millar has been subjected to a course of treatment such as we hope the magistrates are not in the habit of adopting in their judicial capacity. Possibly there may have been ample ground for dismissing Mr. Millar; but it is apparent from the statement of Mr. Millar—backed by the remarks made by Dr. Lee, as recorded in our report of the Quarter Sessions, that the causes of complaint, whatever they were, were never fairly investigated. Here is Mr. Millar's account of the judicial process which resulted in his dismissal:—

"At an ordinary monthly meeting of the Committee, held at the Asylum on the 15th of last August, a statement was made, without notice, by a member of the Committee, who has never been round the Asylum, who has not had any conversation with me respecting its management, and who has only been six months on the Committee. This statement led to the adoption of an abstract resolution to alter the duties of the chief officers. I was then called in, and the resolution was read to me, to which I offered no objection; and I was unexpectedly asked a few questions by the same member of the Committee, with reference to certain matters which appear in the subjoined statement, but without knowing what object he had in view. Portions of my answers were taken down by his direction, and after a very short deliberation in my absence, I was summarily told 'that the Committee had no confidence in my management of the Asylum.' *This was the first and only occasion on which I have had, publicly, or privately, the most remote intimation of the existence of the slightest feeling of dissatisfaction with me.*"

Of course, under such circumstances, Mr. Millar could not but tender his resignation, which was immediately accepted, and though he afterwards wished to withdraw it, he was not allowed to do so. Here the curtain drops ; Mr. Millar is dismissed, and we can only hope that his successor may not be unduly troubled with self-respect, or that he may be more fortunate in conciliating the favour of gentlemen who do not scruple to treat a professional man of high standing in this cavalier manner.

As to the matters alleged against Mr. Millar, we are of course in the dark. As they were not made known to him, of course he cannot inform us what they were. We can only guess at the grounds of dissatisfaction, from the nature of the evidence which Mr. Millar was *entrapped into giving against himself*, which will be found at length in his pamphlet ; and on which we desire to offer no opinion. One of these charges relates to the employment of a patient in the chaplain's garden ; another, and the most serious of all, would lead us to infer that a large discretion had been allowed to the attendants in the administration of the shower-bath. Mr. Millar's observations on these points seem reasonable enough, and if the Committee had chosen to offer any statement on the other side, we might judge between them. It does happen, however, that both these points are prominently referred to in an anonymous letter which has been circulated among the Justices, and which we should imagine to be the production of some underling discharged from the Asylum. This circular we know was read at one of the meetings of the Committee,—we believe at that referred to in the above extract. It must be deemed a very unwise proceeding to take the slightest notice of such a document, and it is not for us to say how much weight it may have had with the Committee. But as they pertinaciously refuse to give any account of their proceedings, the public must be content to suppose that, without any other authority than this trumpery document, they have taken so serious a step as the dismissal, without a hearing, of the chief officer of such an establishment.

If the Justices are wronged by Mr. Millar's pamphlet or by our remarks, they have only themselves to blame. They have been called upon for an explanation by those whose right they can hardly question. It is not merely the ratepayers—seldom mentioned in the court except as the subject of some vulgar jeer—whose curiosity would be gratified by some light on the interior of this costly establishment. It is not merely the "ribald" press that asks for some explanation. The Committee have been challenged in open court by one of their own number to defend themselves from a charge involving gross injustice and folly. They have been told by a magistrate whose official position gives double weight to his remarks, that such a measure would not be dealt out to the porter of a workhouse. In the face of these officials, Mr. Raymond Barker and the rest of the Committee preserved an insulting silence. We firmly believe to be the truth, that the facts of the case if fully known would do much more than confirm the worst suspicions to which so extraordinary a course must give rise.

